# miR-199a-3p plays an anti-tumorigenic role in lung adenocarcinoma by suppressing anterior gradient 2

**DOI:** 10.1080/21655979.2021.1967009

**Published:** 2021-10-10

**Authors:** Hui Liu, Yanfeng Wang, Yi Wang, Daoyuan Wu, He Zhang

**Affiliations:** a Department of Pathology, The Affiliated Cancer Hospital of Zhengzhou University, Zhengzhou, China; b Department of Pathology, Heilongjiang Province Land Reclamation Headquarter General Hospital, Harbin, China

**Keywords:** MIR-199A-3P, AGR2, lung adenocarcinoma, cancer, lung cancer

## Abstract

Previous studies have explored the association between protein-coding genes and microRNAs (miRNAs) in lung adenocarcinoma (LUAD). However, the influence of the miR-199a-3p/anterior gradient 2 (AGR2) axis in LUAD has not yet been fully explored. Therefore, this study aimed to examine the underlying roles of AGR2 and miR-199a-3p in the development of LUAD. The expression levels of miR-199a-3p and AGR2 in LUAD tissues and cells were detected via quantitative reverse transcription-polymerase chain reaction (qRT-PCR). A luciferase assay was also performed to identify the interaction between AGR2 and miR-199a-3p. Moreover, the cell counting kit 8 (CCK-8), 5ʹ-bromo-2ʹ-deoxyuridine (BrdU), and adhesion assays were used along with flow cytometry to verify the malignancy of LUAD in vitro, while a xenograft tumor assay was performed to confirm the tumor growth in vitro. The findings showed a decrease in the expression of miR-199a-3p in LUAD. Additionally, miR-199a-3p overexpression inhibited the growth of LUAD cells in vitro and in vivo, while elevating the apoptosis rate of the cells. AGR2 knockdown had the same effect in the cells as that of miR-199a-3p overexpression. It was also found that miR-199a-3p directly targeted AGR2 in LUAD cells to suppress tumorigenesis. In conclusion, this study suggests that miR-199a-3p plays an anti-tumorigenic role in LUAD by targeting AGR2. Moreover, our study provides insights into the development of novel therapeutic targets for the treatment of LUAD.

## 1. Introduction

Lung cancer is a malignancy that originates in the lungs, which accounted for 1.5 million incident cases in 2017 [1]. There are many types of lung cancer, and lung adenocarcinoma (LUAD) is the most frequent type of lung cancer, accounting for almost 40% of all lung cancer cases [2]. Due to its insidious symptoms, LUAD tends to be diagnosed at a late stage, resulting in a dismal clinical outcome of patients [3]. This disease can be treated by several methods, including surgery, chemotherapy, targeted drug therapy, radiotherapy, and medications. The antitumorigenic potential of fungi-associated materials has also been explored in clinical practice [4]. Although satisfactory clinical outcomes have been achieved with these treatment methods, the 5-year overall survival rate of patients with LUAD remains poor (11%) [5]. Moreover, patients with LUAD often experience a relapse of the disease within the first five years of treatment [5]. By investigating the underlying mechanism of LUAD, it may be possible to improve the strategies for the diagnosis and treatment of LUAD.

MicroRNAs (miRNAs), with 20–23 nucleotides, are endogenous non-coding RNAs that can regulate the expression of target genes [6,7]. MiRNAs can influence various biological processes including cell proliferation, cell apoptosis and cell migration [8–12]. The miRNA microarray analysis led to the identification of five downregulated miRNAs in LUAD samples, including miR-3152, miR-1296, miR-376a, miR-3648, and miR-199a-3p. Among them, miR-199a-3p is associated with several types of cancers, including esophageal, ovarian, and gastric cancers [13–15]. The knockdown of miR-199a-3p can suppress the cell aggressiveness, cell growth, and metastasis by binding to the target genes [14,16,17]. MiR-199a-3p performs various functions, such as blocking the growth and angiogenesis of tumors in hepatocellular carcinoma [18]. MiR-199a-3p is an important indicator for evaluating the prognosis of patients with muscle-invasive bladder cancer after radical cystectomy [19]. Upregulation of miR-199a-3p inhibits the expression of zinc‐finger E‐box-binding homeobox 1 (ZEB1) to restrain the tumor stem-like properties in non-small cell lung carcinoma (NSCLC) cells [20]. Nonetheless, the potential mechanism of action of miR-199a-3p in LUAD needs to be further investigated.

Located on chromosome 7q21.1, the anterior gradient 2 (AGR2) consists of eight exons. AGR2, a member of the protein disulfide isomerase (PDI) family of endoplasmic reticulum (ER) proteins, regulates the growth and metastasis of malignant tumors [21,22]. *AGR2* was also identified as an oncogene in multiple cancers, including the pancreatic, colorectal, and gastric cancers [22–24]. Upregulation of AGR2 is associated with the initiation of cancer, and this protein can induce cell apoptosis and enhance the sensitivity of pancreatic cancer cells to chemotherapy via the extracellular signal‐regulated kinase (ERK)/serine-threonine kinase (AKT) pathways [25]. Furthermore, AGR2 overexpression predicts the poor survival rate in lung cancer [26]. So far, only one study has demonstrated the ability of miR-342-3p to prevent cell growth and migration by inhibiting AGR2 in NSCLC [27]. Furthermore, AGR2 activates the mammalian target of rapamycin complex 2 (mTORC2) pathway and promotes cancer metastasis [28]. Meanwhile, miR-199a-3p retards tumorigenesis in a hepatocellular carcinoma transgenic mouse model by modulating the mTORC2 pathway [29]. Therefore, we hypothesized that *AGR2* may be the key regulator in LUAD and the target gene of miR-199a-3p, which regulates the development of LUAD. The results of this study can help to increase our understanding of the strategies that can be used for the diagnosis and treatment of LUAD.

## 2. Materials and methods

### 2.1 LUAD specimens and cell lines

We obtained 32 paired LUAD tissues and adjacent normal tissues (5 cm from the tumor) from patients with LUAD who consented to participate in this experiment. The collected samples were stored in the TRIzol reagent (cat#: 15,596,026; Invitrogen, USA) at −80°C for RNA extraction. The clinical characteristics of the 32 patients are listed in Supplementary Table S1. All cell lines were purchased from the American Type Culture Collection (USA) and included human LUAD cell lines (PC9, A549, H1975, DV-90, and Calu-3) as well as the normal lung epithelial cell line (BEAS-2B). H1975 and DV-90 cells were maintained in the Roswell Park Memorial Institute (RPMI)-1640 medium, while A549, PC9, BEAS-2B, and Calu-3 cells were transferred to Dulbecco’s modified Eagle medium (DMEM). Then, fetal bovine serum (FBS, 10%) was added to the DMEM or RPMI-1640 media, and the cells were cultured at 37°C in air containing 5% carbon dioxide (CO_2_). All reagents, including the culture medium and FBS, were purchased from Life Technologies Corporation (USA).

### 2.2 Quantitative reverse transcription-polymerase chain reaction (qRT-PCR)

Total RNA from the LUAD tissues, adjacent tissues, LUAD cells, and BEAS-2B cells was isolated using the TRIzol reagent (cat#: 15,596,026; Invitrogen, USA). Reverse transcription was conducted using the PrimeScript First Strand cDNA Synthesis Kit (Cat#: 6210A; Takara, Japan). qRT-PCR was performed using SYBR Premix Ex Taq (Cat#: DRR420A; Takara, Japan). AGR2 expression was normalized to that of glyceraldehyde 3-phosphate dehydrogenase (GAPDH), while the expression of miR-199a-3p was normalized to U6. Finally, data analysis was performed using the 2^−∆∆CT^ method [30]. The primers used in this study are listed in Supplementary Table S2.

### 2.3 Cell transfection

Small interfering RNA (siRNA) negative control (si-NC), si-AGR2, mimic-negative control (mimic-NC), inhibitor-NC, and miR-199a-3p mimics/inhibitor were purchased from RiboBio Co., Ltd (China). Then, PC9 and Calu-3 cells (3 × 10^5^ cells/well) were seeded in 24-well plates and treated with 50 nM siRNA vectors or miR-199a-3p synthetic oligonucleotides using the Lipofectamine 3000 Transfection Reagent (Invitrogen, USA) according to the manufacturer’s protocol. The cells were maintained for 48 h for use in subsequent experiments.

### 2.4 Cell counting kit 8 (CCK-8) assay

The CCK-8 kit (Cat#: K1018) purchased from APExBIO (China) was used to investigate the cell viability. PC9 and Calu-3 cells at a density of 5,000 cells/well were added to 96-well plates. The CCK-8 solution was diluted to a ratio of 1:10 using the medium. At 0, 12, 24, 48, and 72 h, the medium was changed with 100 µL CCK-8 and incubated for 3 h at 37°C. Finally, the optical density (OD) was measured at 450 nm using a microplate reader (Tecan, Switzerland).

### 2.5 5ʹ-bromo-2ʹ-deoxyuridine (BrdU) assay

The BrdU Cell Proliferation Assay Kit (Cat#: 6813; Cell Signaling Technology, USA) was used to perform the BrdU assay. Briefly, PC9 and Calu-3 cells with a density of 2 × 10^4^ cells/well were plated on 96-well plates for 12 h, and an FBS-free medium was used to culture the cells for 12 h. Next, the medium was changed with the BrdU working solution for 4 h, and the mixture was incubated at 37°C. After removing the labeling medium, the cells were fixed and denatured with a fixation/denaturation solution. Next, the BrdU mouse monoclonal antibody (mAb) was added to the solution, and the mixture was cultured for 2 h. Anti-mouse IgG and horseradish peroxidase (HRP)-linked antibodies were subsequently used to detect the binding antibody. Tetramethylbenzidine (TMB), a HRP substrate, was then added to color the mixture. Finally, the optical density (OD) value at 450 nm was measured using a microplate reader (Tecan, Switzerland).

### 2.6 Cell adhesion assay

The cell adhesion plate was first prepared by adding 30 μL/well of the 40 μg/mL collagen I solution (Cat#: C7661; Sigma-Aldrich, USA) to the 96-well plate, and it was stored at 4°C for 12 h. Then, PC9 and Calu-3 cells (2 × 10^4^ cells/well) were plated on the 96-well plates for 24 h. Next, 10 mM ethylenediaminetetraacetic acid (EDTA) (solute in DMEM) was added for 10 min to dissociate the cells. After the cells were suspended in DMEM with 0.1% bovine serum albumin (BSA), 100 μL of the cell suspension was added to the collagen I solution, and the mixture was incubated at 37°C. Non-adherent cells were then washed with PBS for three times, and the cells were cultured at 37°C for 1 h. After 1 h of incubation, the cells were incubated with 10 μL of 3-(4, 5-dimethylthiazol-2-yl)-2, 5-diphenyltetrazolium bromide (MTT) substrate (Cat#: CT01; Sigma-Aldrich, USA) at 37°C for 2 h. Finally, the OD of each well was measured at 570 nm using a microplate reader (Tecan, Switzerland).

### 2.7 Apoptosis assay

The apoptosis rates of PC9 and Calu-3 cells were assessed using the Cell Apoptosis Detection Kit (BD, USA). Cells (6 × 10^4^) were harvested at logarithmic growth phase and suspended in 100 µL of the binding buffer. After that, the cells were incubated with a mixture containing 5 µL/well Annexin V-fluorescein isothiocyanate (FITC) and 5 µL/well propidium iodide (PI) in the absence of light for 15 min at 25°C. After incubation, the cells were washed three times with PBS and suspended in 400 µL of the binding buffer. Flow cytometry was used to detect the cells. The cell apoptosis rate was analyzed by summing the percentage of upper-right and lower-right quadrants.

### 2.8 Xenograft tumor assay

miR-199a-3p agomir and NC were purchased from RiboBio Co., Ltd. (China). BALB/c nude mice (4 weeks old) were purchased from Charles River (Wilmington, MA, USA) and were allocated into two groups, NC and miR-199a-3p agomir. After transfection for 48 h, 2 × 10^6^ Calu-3 cells transfected with miR-199a-3p agomir or its corresponding NC were collected and resuspended using 0.2 mL phosphate-buffered saline (PBS). The transfected cells were then subcutaneously injected into the nude mice. The xenografts were observed after cell inoculation. Five weeks after injection, the nude mice were sacrificed, and the xenografts were removed and photographed. The tumor weight was determined and the tumor samples were stored in liquid nitrogen.

### 2.9 Luciferase assay

The pmiRGLO-AGR2 3ʹ-untranslated region (UTR) wild-type (WT) and mutant (MUT) plasmids were designed and constructed by Tuoran Co., Ltd (Shanghai, China). Next, 5 × 10^5^ PC9 and Calu-3 cells were added to 24-well plates and co-transfected with pmiRGLO-AGR2 WT or MUT plasmids and miR-NC or miR-199a-3p mimic using Lipofectamine 3000. The dual-luciferase reporter assay system was then used to detect the firefly and Renilla luciferase activities after 72 h of transfection. This detection was performed using the Luciferase Assay Kit (Cat#: #16,185; Thermo Scientific, USA), and the firefly luciferase activity was normalized to the results of relative luciferase activity.

### 2.10 RNA pull-down assay

Two biological materials were purchased from Thermo Fisher Scientific (USA). They included biotin-labeled miR-199a-3p (bio-miR-199a-3p) and its negative control (bio-NC). PC9 and Calu-3 cells were first treated with Bio-miR-199a-3p and Bio-NC for 48 h. Subsequently, the cells were collected in a lysis buffer containing RNase and inhibitors. After washing, the streptavidin beads (Cat#: #88,817) from Thermo Fisher (USA) were added to the cell lysates. After the mixture was incubated overnight at 4°C, the RNAs in the beads were eluted and purified using the RNeasy Mini Kit (Cat#: 74,104; QIAGEN, Germany). Finally, qRT-PCR analysis was performed to verify the enrichment of AGR2 in the bio-miR-199a-3p and bio-NC groups.

### 2.11 Statistical analysis

The mean ± standard deviation (SD) of all the data was obtained using the SPSS v.19.0 software (IBM Corp., USA). The paired or unpaired Student’s *t*-test was applied to compare the statistical difference between two groups, while the one-way analysis of variance (ANOVA) followed by Dunnett’s post hoc test was applied to compare the statistical differences among multiple groups. All experiments were performed at least thrice to eliminate errors. Statistical significance was set at P < 0.05.

## 3. Results

### 3.1 MiR-199a-3p and AGR2 were associated with LUAD

**Figure 1.**
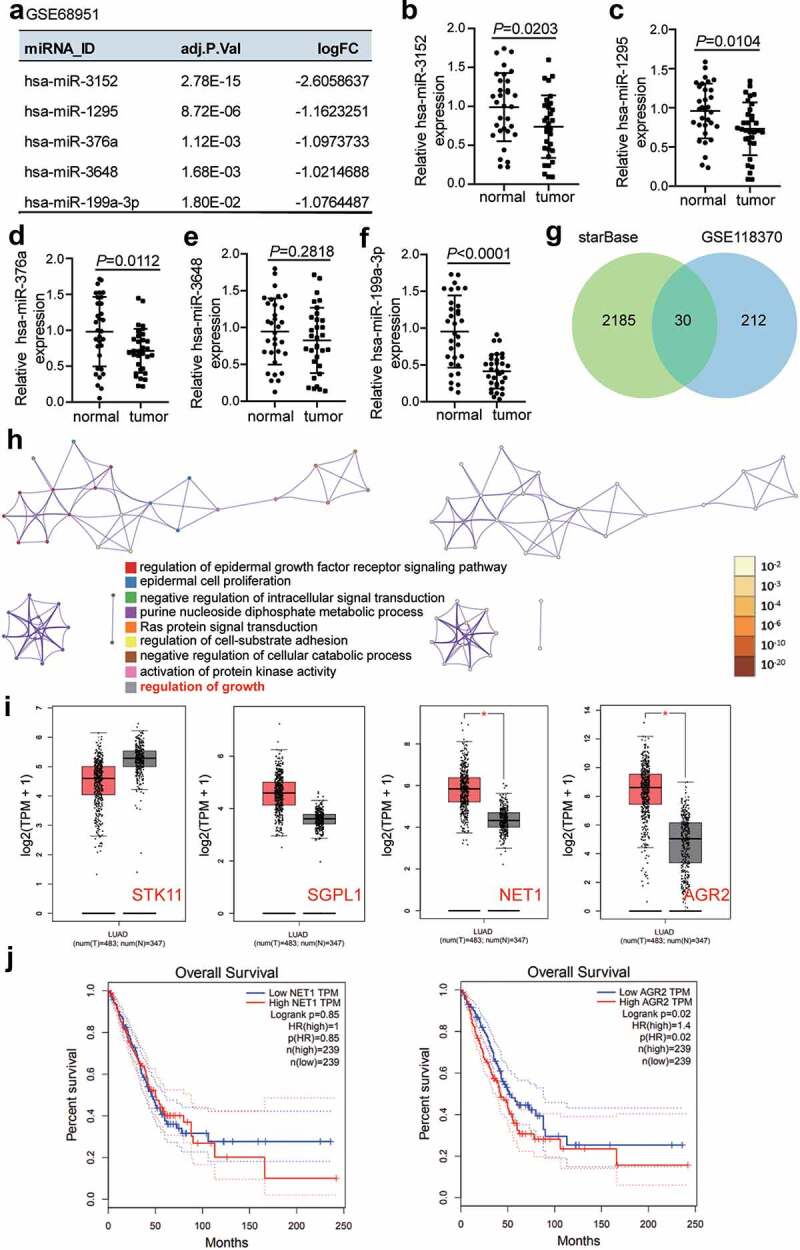
**Bioinformatics analysis identified that miR-119a-3p and AGR2 were associated with LUAD**.(a) Five downregulated miRNAs in LUAD samples were screened from GSE68951 with adj.P value <0.05 and log_2_FC <-1. (b-f) The expression of five downregulated miRNAs in our collected LUAD samples was detected by qRT-PCR. (g) 30 genes were overlapped from GSE118370 and starBase. GSE118370 was a mRNA expression profile to select the upregulate genes in LUAD samples with adj.P value <0.05 and log_2_FC >1. starBase was used to predict the target genes of miR-199a-3p. (h) The regulation of growth was a key biological process by Metascape analysis. Bar graph of enriched terms colored by different biological process (left) and p-values (right). (i) The expression of four genes (STK11, SGPL1, NET1 and AGR2) associated with regulation of growth in LUAD samples based on GEPIA data. Red, tumor group. Gray, normal group. *, *P* < 0.01. (j) The prognosis of NET1 and AGR2 in LUAD was analyzed by GEPIA database

STK11: serine/threonine kinase; SGPL1: sphingosine-1-phosphate lyase 1;NET1: neuroepithelial cell transforming 1; AGR2:anterior gradient 2; LUAD: lung adenocarcinoma; GEPIA: Gene Expression Profiling Interactive Analysis.

GSE68951 [31], an miRNA expression profile obtained from the Gene Expression Omnibus (GEO) datasets (https://www.ncbi.nlm.nih.gov/gds/?term=), including 12 non-tumor lung samples and 203 lung cancer samples. With adj.P-value < 0.05, and log_2_fold change (log_2_FC) <-1, five downregulated miRNAs were screened out from the lung cancer samples compared to non-tumor lung samples. These included miR-3152, miR-1295, miR-376a, miR-3648, and miR-199a-3p (Figure 1a). Among the miRNAs, miR-199a-3p was found to have the lowest expression in LUAD samples compared to adjacent normal samples (Figure 1b-f). GSE118370 [32], an mRNA expression profile downloaded from GEO datasets, includes six normal lung samples and six lung adenocarcinoma samples. It was used to select the upregulated genes in LUAD samples with adj.P-value < 0.05, and log_2_FC >1. To predict the target genes to which miR-199a-3p could bind, starBase (http://starbase.sysu.edu.cn/) was utilized. A total of 30 genes overlapped between GSE118370 and starBase (Figure 1g). After uploading them to Metascape (http://metascape.org/gp/index.html#/main/step1), four genes: serine/threonine kinase 11 (STK11), sphingosine-1-phosphate lyase 1 (SGPL1), neuroepithelial cell transforming 1 (NET1), and AGR2, were observed to be associated with the regulation of growth (Figure 1h). Gene Expression Profiling Interactive Analysis (GEPIA) (http://gepia.cancer-pku.cn/index.html) analysis indicated that NET1 and AGR2 were significantly upregulated in LUAD samples (Figure 1i). The effects of AGR2 on LUAD were explored because its high expression indicated poor prognosis of LUAD based on GEPIA data (Figure 1j).

### 3.2 MiR-199a-3p inhibited the malignancy of LUAD cells both in vitro and in vivo

**Figure 2.**
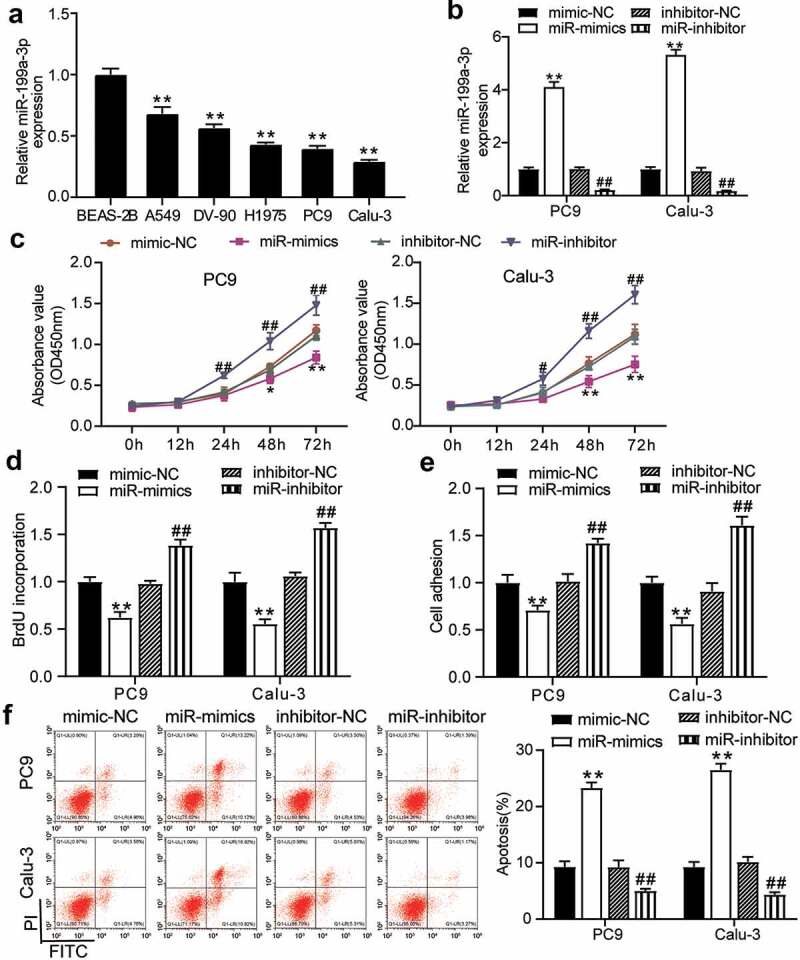
**MiR-199a-3p inhibited cell proliferation and adhesion, and enhanced cell apoptosis in LUAD**.(a) RT-qPCR detection of miR-199a-3p expression in LUAD cells lines (A549, H1975, PC9, and Calu-3) and normal esophageal epithelial cell (BEAS-2B). (b) Measurement of miR-199a-3p expression in PC9 and Calu-3 cells transfected with mimic-NC, and miR-199a-3p mimic, inhibitor NC, and miR-199a-3p inhibitor by RT-qPCR. (c) Cell viability was detected in PC9 and Calu-3 cells transfected with mimic-NC, and miR-199a-3p mimic, inhibitor NC, and miR-199a-3p inhibitor by CCK-8 assay. (d) Cell proliferation was detected in PC9 and Calu-3 cells transfected with mimic-NC, and miR-199a-3p mimic, inhibitor NC, and miR-199a-3p inhibitor. (e) Cell adhesion was detected in PC9 and Calu-3 cells transfected with mimic-NC, and miR-199a-3p mimic, inhibitor NC, and miR-199a-3p inhibitor. (f) Cell apoptosis rate was detected in PC9 and Calu-3 cells transfected with mimic-NC, and miR-199a-3p mimic, inhibitor NC, and miR-199a-3p inhibitor. Data are presented as mean ± SD of at least three independent tests per experiment

**Figure 3.**
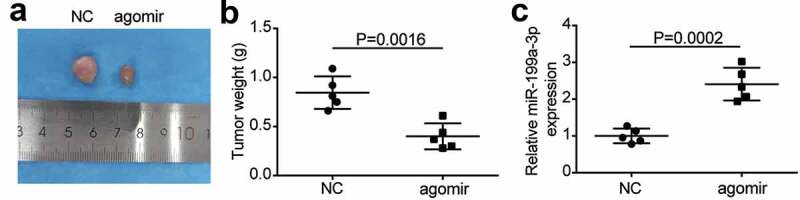
**miR-199a-3p inhibited tumor growth in vivo**.(a) Representative images of tumor growth in NC and miR-199a-3p agomir group. (b) Tumor weight in NC and miR-199a-3p agomir group was counted. (c) The expression of miR-199a-3p in tumor tissues with the transfection of NC or miR-199a-3p agomir was detected by qRT-PCR. NC: Negative control

To examine the function of miR-199a-3p in LUAD cells, the expression levels of miR-199a-3p in LUAD cell lines (PC9, Calu-3, H1975, A549, and DV-90) and normal lung epithelial cells (BEAS-2B) were identified. Experimental results showed that miR-199a-3p expression decreased in LUAD cell lines compared to that in BEAS-2B cells. More specifically, miR-199a-3p expression declined by more than 50% in PC9 and Calu-3 cell lines compared to that in BEAS-2B cells (Figure 2a); therefore, these two cell lines were selected for further research. After transfection with mimics negative control (NC), miR-199a-3p mimics, inhibitor-NC, or miR-199a-3p inhibitor into PC9 and Calu-3 cell lines, results displayed that miR-199a-3p mimics increased by more than 4-fold in miR-199a-3p expression compared to mimic-NC groups; however, miR-199a-3p expression declined by 80% in the miR-199a-3p inhibitor groups compared to the inhibitor NC groups in both PC9 and Calu-3 cell lines (Figure 2b). Furthermore, cell viability was downregulated in the miR-199a-3p mimic group compared to that in the mimic-NC group. However, in both PC9 and Calu-3 cell lines, the miR-199a-3p inhibitor increased cell viability compared to the inhibitor-NC group (Figure 2c). While cell proliferation in the miR-199a-3p mimic groups decreased by 50% compared to the mimic NC groups, cell proliferation in the miR-199a-3p inhibitor groups increased by 1.5-fold compared to the inhibitor NC groups in both PC9 and Calu-3 cell lines (Figure 2d). Cell adhesion in the miR-199a-3p mimic groups declined by 40% compared to the mimic NC groups; nonetheless, cell adhesion in the miR-199a-3p inhibitor groups increased by 1.5-fold in contrast to the inhibitor NC groups in both PC9 and Calu-3 cell lines (Figure 2e). Moreover, the cell apoptosis rate increased by 2-fold in the miR-199a-3p mimic groups, but it declined by 50% in both PC9 and Calu-3 cell lines after transfection with miR-199a-3p inhibitor (figure 2f). Moreover, the effect of miR-199a-3p on LUAD cells was observed in nude mice, showing that miR-199a-3p overexpression inhibited tumor growth (Figures 3a and 3b), and miR-199a-3p agomir enhanced miR-199a-3p expression in tumor sections compared to the NC group (Figure 3c). These results suggest that miR-199a-3p is a tumor suppressor in LUAD.

*, *P* < 0.05; **, *P* < 0.001 compared to mimic-NC; ^#^, *P* < 0.05; ^##^, *P* < 0.001 compared to inhibitor-NC. NC, negative control; mimic, miR-199a-3p mimic; inhibitor, miR-199a-3p inhibitor. LUAD: lung adenocarcinoma; NC: Negative control.

### 3.3 AGR2 was a target of miR-199a-3p

**Figure 4.**
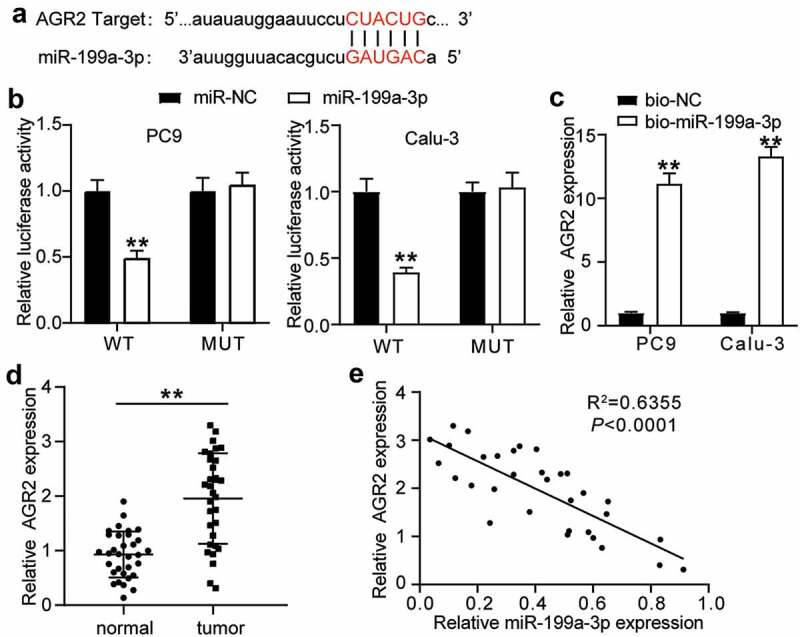
**AGR2 was a target of miR-199a-3p**.(a) starBase showed the predicted binding sequence of AGR2 3ʹ-UTR. (b) Dual luciferase assay was performed in LUAD cells co-transfected with plasmids AGR2-WT or AGR2-MUT and miR-NC or miR-199a-3p mimic in PC9 and Calu-3 cells. (c) RNA pull-down assay identified that the AGR2 expression was pulled down by miR-199a-3p. (d) RT-qPCR detection of expression of AGR2 in the LUAD tumor tissues. (e) Correlation analysis between the miR-199a-3p expression and AGR2 expression in the LUAD tumor tissues. Data are presented as mean ± SD of at least three independent tests per experiment

To further investigate the interaction between AGR2 and miR-199a-3p, the potential binding sites in AGR2 3ʹUTR were predicted using starBase and are shown in Figure 4a. The luciferase assay results indicated that the luciferase activity of the wild-type AGR2 3**′**-UTR decreased by more than 50% in the miR-199a-3p mimic group, but the luciferase activity of the mutant AGR2 3-UTR plasmid showed no difference in both PC9 and Calu-3 cell lines, suggesting that miR-199a-3p was directly attached to the AGR2 3**′**-UTR (Figure 4b). The results of the RNA-pull down assay showed that AGR2 expression was enriched in the Bio- miR-199a-3p group in both PC9 and Calu-3 cell lines (Figure 4c). AGR2 expression increased 2-fold in tumor tissues compared to normal tissues (Figure 4d). As for the outcome of the correlation analysis, a negative correlation between miR-199a-3p expression and AGR2 expression was found in LUAD tissues (Figure 4e). Taken together, these results suggest that miR-199a-3p targets AGR2.

**, *P* < 0.001. WT, wild-type; MUT, mutant; NC, negative control. LUAD: lung adenocarcinoma.

### 3.4 MiR-199a-3p played an anti-tumorigenic role in LUAD by targeting AGR2

**Figure 5.**
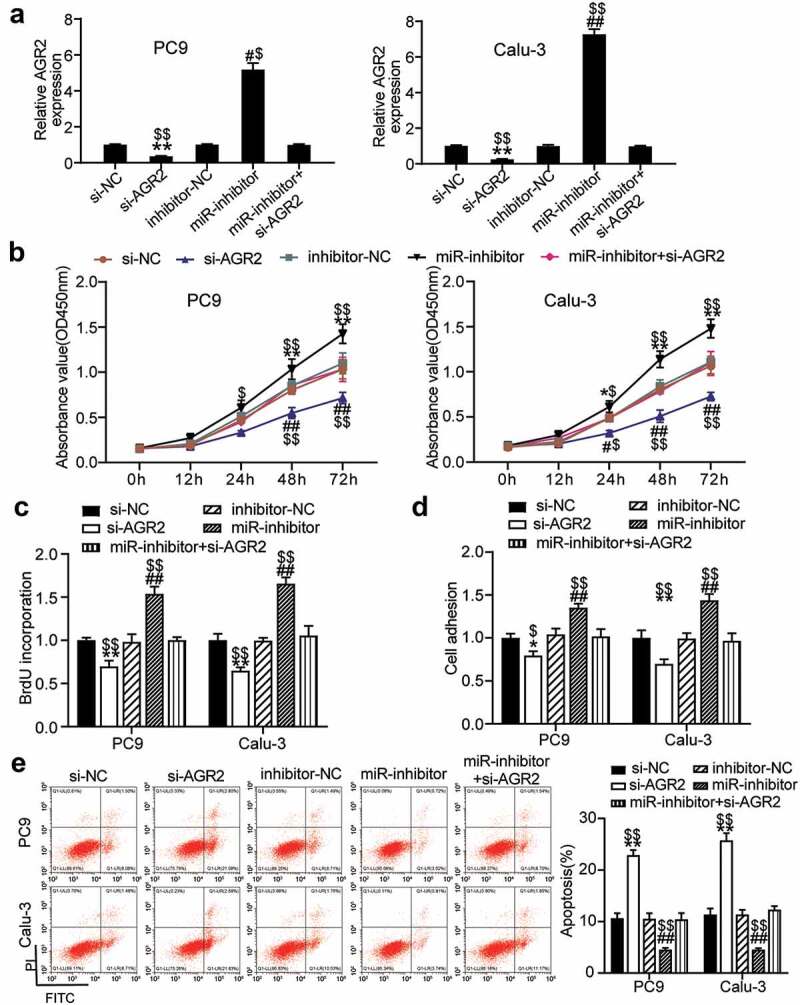
**MiR-199a-3p targeting AGR2 regulated cell proliferation and apoptosis in LUAD**.(a) Measurement of AGR2 protein expression in PC9 and Calu-3 cells transfected with Si- NC, Si-AGR2, inhibitor-NC, miR-199a-3p inhibitor, and Si-AGR2+ miR-199a-3p inhibitor by western blot. (b) Cell viability was detected in PC9 and Calu-3 cells transfected with Si- NC, Si-AGR2, inhibitor-NC, miR-199a-3p inhibitor, and Si-AGR2+ miR-199a-3p inhibitor by CCK-8 assay. (c) Cell proliferation was detected in PC9 and Calu-3 cells transfected with Si- NC, Si-AGR2, inhibitor-NC, miR-199a-3p inhibitor, and Si-AGR2+ miR-199a-3p inhibitor by BrdU assay. (d) Cell adhesion level was determined in PC9 and Calu-3 cells transfected with Si- NC, Si-AGR2, inhibitor-NC, miR-199a-3p inhibitor, and Si-AGR2+ miR-199a-3p inhibitor. (e) Cell apoptosis was determined in PC9 and Calu-3 cells transfected with Si- NC, Si-AGR2, inhibitor-NC, miR-199a-3p inhibitor, and Si-AGR2+ miR-199a-3p inhibitor by FITC apoptosis detection kit. Data are presented as mean± SD of at least three independent tests per experiment

To investigate the mechanism of miR-199a-3p in LUAD, si-NC, inhibitor-NC, Si-AGR2, miR-199a-3p inhibitor, and Si-AGR2+ miR-199a-3p inhibitor were transfected into PC9 and Calu-3 cells. The qRT-PCR results showed that AGR2 expression in the miR-199a-3p inhibitor group increased 5-fold in PC9 cells and 7-fold in Calu-3 cells, whereas AGR2 expression in the Si-AGR2 group decreased by more than 70% in both PC9 and Calu-3 cells. Nonetheless, AGR2 expression in the Si-AGR2+ miR-199a-3p inhibitor group was similar to that in the NC groups (si-NC or inhibitor-NC) (Figure 5a). The cell viability of the Si-AGR2 groups was significantly lower than that of the Si-NC groups; however, this effect was reversed in the Si-AGR2^+^ miR-199a-3p inhibitor groups in both PC9 and Calu-3 cells (Figure 5b). Similarly, the cell proliferation of the Si-AGR2 groups was lower than that of the Si-NC groups; however, this change was restored in the Si-AGR2+ miR-199a-3p inhibitor groups in both PC9 and Calu-3 cells (Figure 5c). In addition, the Si-AGR2 groups showed a 30% downregulation of cell adhesion ability compared to the Si-NC groups; however, the effect was abrogated in the Si-AGR2^+^ miR-199a-3p inhibitor groups in both PC9 and Calu-3 cells (Figure 5d). The results of the cell apoptosis assay indicated that Si-AGR2 enhanced the apoptosis rate by 2-fold compared to the si-NC group; however, the effect was reversed in the Si-AGR2+ miR-199a-3p inhibitor groups in both PC9 and Calu-3 cells (Figure 5e). Overall, the data indicated that by targeting AGR2, miR-199a-3p repressed cell proliferation but induced cell apoptosis in LUAD.

*, *P* < 0.05; **, *P* < 0.001 compared to Si-NC; ^#^, *P* < 0.05; ^##^, *P* < 0.001 compared to inhibitor-NC; ^$^, *P* < 0.05; ^$$^, *P* < 0.001 compared to miR-inhibitor+si-AGR2. Si-NC, SiRNA-AGR2 negative control; inhibitor-NC, miR-199a-3p inhibitor negative control; miR-inhibitor, miR-199a-3p inhibitor; Si-AGR2, SiRNA-AGR2; Si +inhibitor, Si-AGR2+ miR-199a-3p inhibitor.

## 4. Discussion

In the present study, we found that miR-199a-3p was less expressed in LUAD tissues and cells, and miR-199a-3p mimic retarded tumor growth in vitro and in vivo, while miR-199a-3p inhibitor strengthened the proliferative capacity of LUAD cells. Mechanistically, miR-199a-3p targeting argonaute 2 (AGO2) inhibited the LUAD progression. These results suggest that targeting the miR-199a-3p/AGO2 axis might be an effective approach for LUAD management.

MiR-199a-3p can regulate cell growth, differentiation, and death in various cancer types, such as esophageal, gastric, and prostate cancers [16,33,34]. MiR-199a-3p significantly inhibits the cell proliferation by interacting with p21-activated kinase 4 in esophageal cancer [35]. MiR-199a-3p also promotes cell invasion and migration in gastric cancer cells, and the upregulation of miR-199a-3p predicts poor prognosis [15]. Moreover, there is increased expression of miR-199a-3p in patients with lung cancer, and this miRNA could serve as a biomarker for diagnosing lung cancer [36,37]. Bai et al. demonstrated that the upregulation of miR-199a-3p inhibited ZEB1 inhibited lung cancer growth in vitro and in vivo [20]. Therefore, miR-199a-3p plays a tumor-suppressing role during cancer progression. Consistently, this study showed that miR-199a-3p expression obviously decreased in LUAD, and miR-199a-3p was found to impair cell growth and promote cell apoptosis in vivo and in vitro. Our findings support previous findings that miR-199a-3p plays a tumor-suppressive role.

The impact of protein-coding genes has been documented in cancer literature. Previous studies have suggested that AGR2 promotes cancer occurrence by increasing cellular growth and downregulating cell apoptosis in different types of cancers [23,38,39]. For instance, Zhang et al.revealed that AGR2 enhanced doxorubicin tolerance in breast cancer cells [38]. FOXA2-AGR2, a key pathway, contributes to the proliferation and tumorigenesis of pancreatic cancer [40]. Although many studies have suggested that AGR2 could serve as a potential biomarker in LUAD progression, the underlying mechanisms remain elusive [41–43]. Similar to the investigations on AGR2 in LUAD, this study found that AGR2 expression increased in LUAD tissues and that AGR2 knockdown hampered cell growth and enhanced cell apoptosis in LUAD cell lines. Generally, miRNA recognition of the 3′-UTR mRNA plays a critical role in gene expression regulation. A previous study also found that miR-342-3p inhibited AGR2 expression, thereby restricting the proliferation and migration of NSCLC cells [27]. Herein, a predicted targeting correlation between the expression of AGR2 and miR-199a-3p was also validated by luciferase reporter assays. Furthermore, AGR2 forced expression almost abolished the inhibitory effect of miR-199a-3p on LUAD cell proliferation and apoptosis. In addition to our findings, Bai et al. demonstrated that the upregulation of miR-199a-3p inhibited ZEB1 expression in lung cancer cells and further confirmed this effect in xenograft mice [20]. MiR-199a-3p is considered to target different genes to interfere with the progression of lung cancer. In future studies, we aim to investigate the complex regulatory network of miR-199a-3p.

## 5. Conclusion

Our findings demonstrate that miR-199a-3p is downregulated in LUAD tissues and cells, and its overexpression reduces the proliferation of LUAD cells, while triggering the apoptosis of cells. Mechanical research has shown that miR-199a-3p influences the malignancy of LUAD by targeting AGR2. The outcome of this research could provide more insights into the development of novel therapeutic targets for the treatment of LUAD. However, this study has several limitations such as Lack of study on downstream signaling pathway. In the future, our study will explore other potential signaling pathways that influence the miR-199a-3p-AGR2 axis in LUAD cells. In addition, the results of this research should be verified with more clinical samples and in vivo assays in the future.

## Supplementary Material

Supplemental Material

## Data Availability

The datasets used and/or analyzed during the current study are available from the corresponding author on reasonable request.

## Highlight

1. MiR-199a-3p overexpression inhibited the growth of the LUAD cells in vitro.

2. MiR-199a-3p retarded the LUAD tumorigenesis in vivo.

3. AGR2 is the target of miR-199a-3p.

## Abbreviations

Lung adenocarcinoma (LUAD); MicroRNAs (miRNAs); non-small cell lung cancer (NSCLC); Anterior gradient 2 (AGR2); disulfide isomerase (PDI); endoplasmic reticulum (ER); log2Fold Change (log2FC); negative control (NC); Si- negative control (si-NC); mimic-negative control (mimic-NC); standard deviation (SD).

## Declarations

**Ethics approval and informed consent**

The present study was approved by the Ethics Committee of the Affiliated Cancer Hospital of Zhengzhou University. The processing of clinical tissue samples is in strict compliance with the ethical standards of the Declaration of Helsinki. All patients signed written informed consent. The processing of animals is in strict compliance with the Ethics Committee of the Affiliated Cancer Hospital of Zhengzhou University. Animal licenses are included in the supplementary files.
