# Supplementary material for: miR-199a-3p plays an anti-tumorigenic role in lung adenocarcinoma by suppressing anterior gradient 2
Source: Bioengineered. 2021 Oct 10;12(1):7859–71. doi: 10.1080/21655979.2021.1967009 (PMC8806604; doi:10.1080/21655979.2021.1967009)
Supplement: Supplemental Material [file KBIE_A_1967009_SM0880.zip › suppl/Supplementary_Table_1_revised.docx]

Supplementary Table 1. The clinical characteristics of 32 LUAD patients.

| Variables | No. (n=32) | percentage (%) |
| --- | --- | --- |
| Median age (range) | 57(34-86) |  |
| Gender |  |  |
| Male | 17 | 52.1 |
| Female | 15 | 47.9 |
| Smoking status |  |  |
| Yes | 19 | 59.4 |
| No | 13 | 40.6 |
| p-T Status: |  |  |
| T1a | 20 | 62.5 |
| T1b | 9 | 28.1 |
| T2a | 3 | 9.4 |
| p-N Status |  |  |
| N0 | 27 | 84.4 |
| N1 | 5 | 15.6 |
| Treatment history |  |  |
| Yes | 14 | 43.8 |
| No | 18 | 56.2 |
