# Supplementary material for: miR-199a-3p plays an anti-tumorigenic role in lung adenocarcinoma by suppressing anterior gradient 2
Source: Bioengineered. 2021 Oct 10;12(1):7859–71. doi: 10.1080/21655979.2021.1967009 (PMC8806604; doi:10.1080/21655979.2021.1967009)
Supplement: Supplemental Material [file KBIE_A_1967009_SM0880.zip › suppl/Supplementary_table_2_revised.docx]

Supplementary Table 2. The primer sequences used in this study.

| primer | | Sequences (5’-3’) |
| --- | --- | --- |
| hsa-miR-3152 | Forward | TGTGTTAGAATAGGGGCA |
|  | Reverse | CAGTGCGTGTCGTGGAGT |
| hsa-miR-1295 | Forward | GCCGCTTAGGCCGCAGATCT |
|  | Reverse | GTGCAGGGTCCGAGGT |
| hsa-miR-376a | Forward | ATTAATCATAGAGGAAATCCACG |
|  | Reverse | GTGCAGGGTCCGAGGT |
| hsa-miR-3648 | Forward | CACGCAGCCGCGGGGAT |
|  | Reverse | CCAGTGCAGGGTCCGAGGTA |
| hsa-miR-199a-3p | Forward | GGTGCAGGGT CCGAGGTAT |
|  | Reverse | GGCGGACAGT AGTCTGCACAT |
| AGR2 | Forward | AGTTTGTCCTCCTCAATCTGGTTT |
|  | Reverse | GACATACTGGCCATCAGGAGAAA |
| GAPDH | Forward | AGCCACATCGCTCAGACAC |
|  | Reverse | GCCCAATACGACCAAATCC |
| U6 | Forward | GCTTCGGCAGCACATATACTAAAAT |
|  | Reverse | CGCTTCACGAATTTGCGTGTCAT |
